# Dust and Noise as a Cause of Disease

**Published:** 1905-02

**Authors:** 


A Monthly Review of Medicine and Surgery.

*EDITOR:*

WILLIAM WARREN POTTER, M. D.

All communications, whether of a literary or business nature, books for review and exchanges, should be addressed to the editor: 284 Franklin St., Buffalo, N. Y.

Vol. XLIV.—LX. FEBRUARY, 1905. No. 7

### Dust and Noise as a Cause of Disease.

IN great cities the medical profession has come to understand that dust and noise are causes that operate importantly in creating disease and in preventing its cure. Dust causes and aggravates diseases of the respiratory tract; noise causes and aggravates disease of the nervous system.

Dust containing disease germs is licked up by the wind and is respired by the throngs that pass along the streets. Just in proportion as a city maintains cleanliness in its avenues of traffic and pleasure, will the disease rate be reduced or maintained at a minimum insofar as respiratory diseases are concerned. The best method of securing clean streets may be regarded as still undetermined; but there is one method which the street cleaning commissioner in New York, Dr. John McGaw Woodbury, has adopted that appeals to common sense as possessing scientific value. We refer to disinfecting the gutters and piles of dirt with a germicide solution before removal. Dusty air causes consumption and weakens lung power if it contains disease germs. Dust, therefore, should not only be reduced to a minimum, but its disease creating power should be destroyed if possible, before it is taken up by the atmosphere, subsequently to be inspired by the people.

If, as has been asserted by competent authority, persons in sound health do not furnish a suitable culture medium for disease germs, then it is very important to keep the population in good health,—to foster resisting power by every reasonable means. How can that be done unless they are prevented from breathing pathogenic bacteria into their lungs? We suggest this as an important question to be taken up by our health authorities.

It has been asserted that the influence of noise as a cause of disease is quite as great as that of dust; the difference being that dust poisons the system through the respiratory tract, while noise manifests its deleterious influence through the nervous system. In health, noise causes irritation and pain, disturbs digestion, prevents sleep and depresses the physical and mental powers, reducing the normal resistance to disease. In sickness, noise simply becomes an abomination, aggravating symptoms, retarding cure, and in many instances absolutely preventing recovery.

The worst feature of all this is, that most of the noises that produce disease or prevent recovery belong to the preventable class. In these days of clocks and watches there is small need of factory whistles and church bells to announce the time for labor, refreshment, or religious services. Such methods are archaic and do not belong to the fashions which should prevail in cities at the present day. If we should undertake to make a catalogue of noises which are, to say the least, needless and wholly within the range of prevention, the list would be a long one. It would embrace noises created by screaming and whistling and those made by cats, dogs, chickens, cows, newsboys, hucksters and scissors grinders, and the noises made in beating carpets, chopping kindling wood, cutting iron, rattling coal through steel chutes, and other unnecessary noises, which could easily be stopped; these and many others which we need not mention at this time.

In Buffalo, the screeching of the fire tug and the bellowing of the fog horn are abominations that ought to make every official ashamed, from the mayor down to the distinguished citizens who pull open the throttles of these two destroyers of peace and health.

The men who control factories, drive locomotives, attend stationary engines, manipulate fire tugs, and deal with other noise producing machinery are, themselves, indifferent to the noises they create and are careless of the comfort and welfare of their fellow citizens. Unless they are controlled by the iron hand of power and law, they will never abandon their propensities to create noise and confusion.

Perhaps it would be wise to organise a society for the prevention of noise and the suppression of dust. Not until some such powerful organisation is created that shall have for its purpose the enlightenment of the community and, above all, the awakening of the city officials from their indifferent lethargy concerning these matters, will the citizens obtain relief.

Since writing the foregoing, another side of the dust problem has been presented to us, through the action of a prominent railway in the adoption of a new method in cleaning its cars.

We could wish also that it would suppress the Pullman porter with his dust brush and pan, and whisk broom, who makes life wearisome and the air almost unbreathable for the parlor car passenger. We print the article referred to entire as an addendum to this presentation of the dust and noise problem:

### ADVANCED METHOD OF REMOVING GERMS AND DUST FROM RAILWAY CARS.

The management of the Central Railroad of New Jersey has made another step of advancement through the recent installation of a system of car cleaning which has the universal approval of the health authorities along its line, and as it is practically the first transportation company to adopt it, the method may be of interest to our readers.

The old method of car cleaning with a whisk here and a dash there with a broom or duster, was not only unsanitary, but unsatisfactory, for the reason that it had the effect largely of removing dust and dirt from one section, and depositing it elsewhere; but under the new method, which is termed the Vacuum Sweeping System, the dirt and dust is drawn from the car by suction through a pipe, and is gone forever. The New Jersey Central has erected an immense vacuum plant in its Jersey City yards, and for a distance of 3,600 feet has laid pipe, varying from two to five inches in diameter, covering in all about three miles. At short intervals this pipe is tapped and from these cocks is run the flexible hose, which may be taken in the car either by door or window. At the foot of the hose is a metal pipe with a flat triangular end, along the base of which is an opening, and through which the dust and dirt is drawn by the vacuum or "drawing-in machine" located a distance away. The operator runs the slot opening over the cushions, carpets, curtains, wood-work, etc., and without any commotion or dust raising, every loose particle or germ is whisked away, everything being left clean and wholesome. The dust thus removed, before reaching the great "drawing-in machine" must pass through two dust separators, the first of which clears the air of 90 per cent. of the grit, dust and germs; the second separator or cylinder draws the air through water in which corrosive sublimate is used, and completes perfectly the purification. The New Jersey Central management has for a long time felt the necessity for a more sanitary method of car cleaning, and the vacuum system, while reducing disease liabilities to a minimum, at the same time reduces the cost of cleaning and time consumed. Two cars can be thoroughly cleaned under the new system at the same expense of time and money as was formerly consumed in cleaning one, and this in connection with the increased sanitary value, is sure to cause its general introduction within a short time, not only by other transportation companies, but by theatres, hotels, places of public resort and even the home.

In still further amplification of this subject we present the following from *American Medicine,* January 21, 1905:

SCIENTIFIC STREET CLEANING.

An example of what can be accomplished by honest endeavor, controlled by scientific knowledge, in the administration of affairs concerning the public is furnished by the work of Street Cleaning Commissioner John M. Woodbury, of New York. Not satisfied with the old and inefficient method of cart-sprinkling and sweeping, he introduced the plan of washing the streets by means of compressed air machines, or with hose from the hydrants. Instead of laying the dust by sprinkling, he says the streets should be washed so clean there will be no dust. During the past year an average of sixty miles of street has been washed daily, between the hours of one and four in the morning. By washing, Dr. Woodbury means applying the water with sufficient force to remove the gum which clings to the surface of asphalt; this, he says, is the only sanitary way to clean such pavement. The proof of his assertion is found not only in the comparative freedom from dust as raised by the older methods, but also in a more positive way of bacteriologic tests. The latter show that bacteria are very largely removed from the streets by washing them as described. An agar plate exposed at a point on Fifth avenue just after the passage of a sprinkling wagon developed 460 colonies of bacteria. A second plate, exposed at the same place for an equal time after approved flushing of the street, showed only 10 colonies. Another proof of the efficacy of the plan adopted is the low death rate in the part of the city which has been so cleaned for a considerable period of time. Extended comment upon these facts would be superfluous. They are made possible by putting the right man in the right place, a consummation devoutly to be desired in many of our graft-cursed cities.

